# Changes of Acute Kidney Injury Epidemiology during the COVID-19 Pandemic: A Retrospective Cohort Study

**DOI:** 10.3390/jcm11123349

**Published:** 2022-06-10

**Authors:** Pasquale Esposito, Elisa Russo, Daniela Picciotto, Francesca Cappadona, Yuri Battaglia, Giovanni Battista Traverso, Francesca Viazzi

**Affiliations:** 1 Unit of Nephrology, Dialysis and Transplantation, Department of Internal Medicine, University of Genoa, 16132 Genoa, Italy; lisa24russo@gmail.com (E.R.); daniela.picciotto@hsanmartino.it (D.P.); cappadona.francesca@gmail.com (F.C.); giovannibattista.traverso@hsanmartino.it (G.B.T.); francesca.viazzi@unige.it (F.V.); 2 IRCCS Ospedale Policlinico San Martino, 16132 Genova, Italy; 3 Department of Medicine, University of Verona, 37134 Verona, Italy; yuri.battaglia@univr.it; 4 Nephrology and Dialysis Unit, Pederzoli Hospital, 37019 Peschiera del Garda, Italy

**Keywords:** acute kidney injury, COVID-19, SARS-CoV-2, hospitalization, mortality

## Abstract

To evaluate the impact of the Coronavirus Disease-19 (COVID-19) pandemic on the epidemiology of acute kidney injury (AKI) in hospitalized patients, we performed a retrospective cohort study comparing data of patients hospitalized from January 2016 to December 2019 (pre-COVID-19 period) and from January to December 2020 (COVID-19 period, including both severe acute respiratory syndrome coronavirus 2 (SARS-CoV-2)-negative and positive patients). AKI was classified by evaluating the kinetics of creatinine levels. A total of 51,681 patients during the pre-COVID-19 period and 10,062 during the COVID-19 period (9026 SARS-CoV-2-negative and 1036 SARS-CoV-2-positive) were analyzed. Patients admitted in the COVID-19 period were significantly older, with a higher prevalence of males. In-hospital AKI incidence was 31.7% during the COVID-19 period (30.5% in SARS-CoV-2-negative patients and 42.2% in SARS-CoV-2-positive ones) as compared to 25.9% during the pre-COVID-19 period (*p* < 0.0001). In the multivariate analysis, AKI development was independently associated with both SARS-CoV-2 infection and admission period. Moreover, evaluating the pre-admission estimated glomerular filtration rate (eGFR) we found that during the COVID-19 period, there was an increase in AKI stage 2–3 incidence both in patients with pre-admission eGFR < 60 mL/min/1.73 m^2^ and in those with eGFR ≥ 60 mL/min/1.73 m^2^ (“de novo” AKI). Similarly, clinical outcomes evaluated as intensive care unit admission, length of hospital stay, and mortality were significantly worse in patients admitted in the COVID-19 period. Additionally, in this case, the mortality was independently correlated with the admission during the COVID-19 period and SARS-CoV-2 infection. In conclusion, we found that during the COVID-19 pandemic, in-hospital AKI epidemiology has changed, not only for patients affected by COVID-19. These modifications underline the necessity to rethink AKI management during health emergencies.

## 1. Introduction

Coronavirus Disease-19 (COVID-19), caused by the infection of severe acute respiratory syndrome coronavirus 2 (SARS-CoV-2), is a complex and multisystemic disease [1].

Indeed, while pulmonary alterations were recognized as the first manifestations of the infection, it is now clear that multiorgan damage may occur, leading to high morbidity, hospitalization rate and mortality [2]. Clinical experience and reports have shown that kidney involvement, defined as both urinary abnormalities and impairment of renal function, is very frequent in patients with COVID-19 (up to 75% of the patients) [3]. In particular, the development of acute kidney injury (AKI) has emerged as a common complication of COVID-19, affecting between 17% and 37% of hospitalized patients [4]. In these patients, it was reported that intrahospital AKI is associated with an increased risk of death during a hospital stay [5]. Multiple reasons may explain the high incidence of AKI in COVID-19 patients, such as hemodynamic alterations, enhanced inflammatory status, coagulation abnormalities, organ cross-talk or a direct renal localization of the SARS-CoV-2 [6]. However, it is now clear that the pandemic, apart from the problems directly correlated to the management of COVID-19 patients, also had relevant indirect effects on society, economics and the general health system organization. In particular, the necessity to face the COVID-19 pandemic required a complete rethinking of health systems and territorial and hospital care. Interesting research conducted by the WHO reported that during the first months of the pandemic there was a worldwide disruption of services for the prevention and treatment of non-communicable diseases, including hypertension and diabetes [7]. Therefore, it is conceivable that the modifications of the usual medical care also had an influence on the clinical management and outcomes of diseases not directly related to COVID-19. In this regard, a recent large retrospective study on a French cohort of hemodialysis patients (HD) showed that the quality of HD delivered to COVID-19-free HD patients was negatively impacted because of the strong constraints on Nephrology units’ organization related to the pandemic [8]. Consistently, in this study, we investigated whether the COVID-19 pandemic could have had an impact on intrahospital AKI incidence and outcomes. For this purpose, we collected data on AKI epidemiology in adult patients hospitalized during the COVID-19 pandemic, including both SARS-CoV-2-positive and negative patients, in a tertiary Hospital, located in the North of Italy. These data were compared with a historical cohort of patients admitted to the same hospital during the four years before the pandemic.

## 2. Materials and Methods

### 2.1. Setting and Study Population

We performed a retrospective observational study on the hospitalized population admitted to Policlinico Universitario San Martino, Genova, Italy. We collected data on adult patients (age > 18 years) admitted from 1 January 2016 to 31 December 2020, splitting this time into two intervals, i.e., pre-COVID-19 period (from 1 January 2016 to 31 December 2019) and COVID-19 period (from 1 January 2020 to 31 December 2020). Among patients admitted during the COVID-19 period, we differentiated patients affected by COVID-19, i.e., tested positive by polymerase chain reaction (PCR) for SARS-CoV-2 on a nasopharyngeal swab (SARS-CoV-2-positive patients) and patients with a negative PCR (SARS-CoV-2-negative). Study patients were included at the time of their first hospital admission. If a patient was hospitalized multiple times during the study period, we considered only the first one. Patients with chronic kidney disease (CKD) stage 4–5 (i.e., estimated glomerular filtration rate (eGFR) < 30 mL/min per 1.73 m^2^) and HD, as identified by ICD-9-CM (International Classification of Disease, 9th Revision, Clinical Modification) diagnosis codes, were not included in the research algorithm. Patients with a length of hospital stay > 30 days were excluded. Our institutional review board approved the protocol (N. Registro CER Liguria: 515/2020) and waived the need for informed consent. The study was also registered on clinicaltrial.gov website (NCT05151003). The research was conducted in accordance with the Helsinki Declaration.

### 2.2. Data Collection

All data were extracted from the hospital’s electronic database. We exported the following demographic, clinical and laboratory data: age, sex, serum creatinine (sCr), intensive care unit (ICU) admission, length of hospital stay, death, comorbidities and primary diagnosis codes at hospital discharge. Comorbid conditions, such as discharge diagnosis, were identified using ICD-9-CM codes (diabetes: ICD 9 CM 250.1–250.7; cardiovascular diseases: ICD 9 CM 390–459; haematological disorders: ICD 9 CM 280–289; malignancies: ICD 9 CM 140–239; infectious disease ICD 9 CM 001–139; respiratory diseases ICD 9 CM 460–519; and so on) [9]. The sCr levels were collected at admission, at discharge, at the lowest and peak levels during the hospitalization.

### 2.3. Definitions

Changes in sCr were calculated by comparing the peak sCr to the lowest sCr during hospitalization under the assumption that the lowest sCr would represent baseline kidney function. We defined AKI according to the *Kidney Disease: Improving Global Outcomes (KDIGO) Clinical Practice Guideline*, based on changes in sCr [10]. We reported each stage according to the KDIGO framework as stages 1, 2, and 3. These correspond to 1.5 to 1.9 times their baseline creatinine, 2 to 2.9 times their baseline creatinine, and 3 or more times their baseline creatinine or newly required dialysis. Urine output was not considered due to the limited habit of collecting this information in routine inpatient care in this hospital.

In patients in whom previous sCr values, measured up to 180 days before the hospitalization, were available, we calculated eGFR, using the Chronic Kidney Disease Epidemiology Collaboration creatinine-based equation [11]. In this subgroup of patients, we distinguish patients with pre-admission eGFR < or ≥ of 60 mL/min/1.73 m^2^, to evaluate the incidence of “de novo” AKI, defined as AKI occurring in patients with pre-admission eGFR ≥ 60 mL/min/1.73 m^2^.

### 2.4. Outcomes and Covariates

The primary and secondary outcomes were incident in-hospital AKI and overall mortality, respectively. Information about cause of death was obtained from hospital records or death certificates.

### 2.5. Statistical Analysis

Normally distributed variables are presented as mean ± SD and compared using an independent or paired *t*-test as appropriate. Logarithmically transformed values of skewed variables were used for the statistical analysis. Comparisons between groups were made by analysis of variance (ANOVA) with post-hoc testing.

Benjamini–Hochberg calculation was used to correct false discovery rates. Supposing to accept a 15% of false discovery rate, we calculated the Benjamini–Hochberg critical value for each *p*-value, using the following formula: (i/6) × 0.15 where i = rank of *p*-value.

Comparisons of proportions were made using the χ2-test or Fisher’s exact test when appropriate. The incidence rate of AKI was calculated. Univariate and multivariate logistic regression analyses were used to describe the relationship between all available clinical variables of biological relevance and the presence of AKI. Odds ratios and 95% confidence intervals were calculated by exponentiation of logistic regression coefficients. Time to event analyses were performed using: (i) Kaplan–Meier method for survival curves estimation and log-rank test to compare them; (ii) univariate and multivariate Cox regression models: risk was reported as hazard ratios (HR) along with their 95% confidence intervals (CI). Covariates included all available clinical variables with biological plausibility. Time variable was defined as the interval time between baseline date and the date of endpoint occurrence or the last available follow-up. Power analysis showed that the number of individuals in the database (*n* = 61,743) represented a sample largely sufficient to avoid 𝛽 error also after stratification by AKI, COVID-19 period and SARS-CoV-2 infection. Statistical calculations were performed by STATA package, version 14.2 (StataCorp, College Station, TX, USA). The null hypothesis was rejected for values of *p* < 0.05.

## 3. Results

### 3.1. Patient General Characteristics

We collected data on 61,743 patients, i.e., 51,681 in the four years constituting the pre-COVID-19 period and 10,062 admitted to the hospital in the COVID-19 period, respectively. Among patients of the COVID-19 period, 9026 patients (89.7%) were SARS-CoV-2-negative, while 1036 (10.3%) were SARS-CoV-2-positive. Overall, compared with the historical cohort of the pre-COVID-19 period, patients admitted during the COVID-19 period were significantly older, with a higher prevalence of males. Moreover, looking at comorbidities identified using reported administrative data in the COVID-19 period, we observed a significant reduction of chronic conditions, such as diabetes, hypertension, cardiac failure, and atrial fibrillation (Table 1). These data were also confirmed when we separately compared pre-COVID-19 patients with patients admitted during the COVID-19 period according to the positivity to SAR-CoV2 infection. However, SARS-CoV-2-positive patients, compared with SARS-CoV-2-negative patients admitted in the same period, were significantly older and with a higher prevalence of hypertension and cardiovascular disease. Of note, opposite findings were observed when looking at the prevalence of malignancies that, during the pandemic period, showed an increase only in SARS-CoV-2-negative patients.

Then, to better characterize the patients we looked at the primary discharge diagnosis. (Supplementary Table S1). Overall, in the COVID-19 period, as expected, there was a significant increase in diagnoses related to respiratory disorders, whereas the diagnosis codes related to cardiovascular disease, hematological disorders, psychiatric diseases, and other infectious diseases decreased. These modifications were partially significant for all the patients admitted during the COVID-19 period, regardless of SARS-CoV-2 infection status.

### 3.2. Kidney Function Time Course: AKI Incidence and Determinants

**Figure 1:**
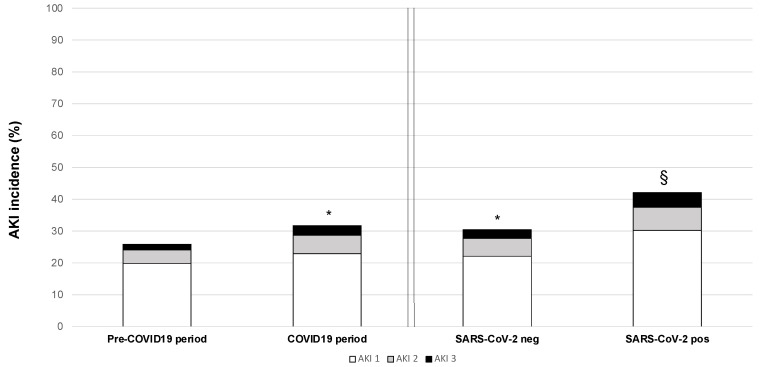
**Incidence and staging of in-hospital AKI**. Comparison of AKI incidence and stages between patients hospitalized in the pre-COVID-19 period (2016–2019) vs. COVID-19 period (2020). Patients of the COVID-19 period were also divided according to SARS-CoV-2 positivity to nasopharyngeal swab. * *p* < 0.0001 vs. pre-COVID-19; § *p* < 0.0001 vs. pre-COVID-19 and SARS-CoV-2 negative. Abbreviations: AKI—acute kidney injury; COVID-19—Coronavirus Disease-19; SARS-CoV-2—severe acute respiratory syndrome coronavirus 2. The different groups were compared by ANOVA with post-hoc testing.

At the admission, patients in the COVID-19 period presented significantly higher sCr compared to the pre-COVID-19 period (Table 2). AKI, defined and graded according to the sCr levels collected during hospitalization, was more common and severe in patients in the COVID-19 period compared to pre-COVID-19 (Table 2 and Supplementary Table S2). These findings were also confirmed when comparing pre-COVID-19 and SARS-CoV-2-negative patients. However, SARS-CoV-2-positive patients presented the highest prevalence of AKI, with an increased number of patients in AKI stage 3 (Figure 1). In 22,042 patients (18,785 in pre-COVID-19 (i.e., 36% of the total patients admitted in the pre-COVID-19 period) and 3257 in the COVID-19 period (i.e., 30.6% of the patients admitted in the COVID-19 period)), we had at least one sCr level measured within six months before the hospitalization. Considering these values, we calculated eGFR and distinguished between the incidence of AKI occurring in patients with pre-admission eGFR < 60 mL/min/1.73 m^2^ and patients experiencing AKI who presented a pre-admission eGFR ≥ 60 mL/min/1.73 m^2^ (“de novo” AKI). SARS-CoV-2-positive patients showed a significantly higher prevalence of patients with pre-admission eGFR < 60 mL/min/1.73 m^2^ and a higher incidence of both AKI occurring in this population and “de novo” AKI compared with pre-COVID-19. At variance, SARS-CoV-2-negative patients showed only a higher incidence of “de novo AKI” as compared to patients hospitalized in the pre-COVID-19. Clinical determinants of in-hospital AKI were analyzed in the entire cohort of patients (Table 3). In both univariate and multivariate analysis, demographic characteristics (age, male sex), length of hospital stay, ICU admission, main comorbidities, basal sCr, admission period (pre-COVID-19 or COVID-19), and SARS-CoV-2 infection were significantly associated with the occurrence of AKI. In particular, the admission during the COVID-19 period increased the risk of AKI (OR 1.18, IC 1.12–2.25) regardless of SARS-CoV2 infection. However, in SARS-CoV-2-positives, the risk of developing AKI was further increased (OR 1.30, IC 1.12–1.5). Finally, we found that patients admitted during the COVID-19 period (particularly SARS-CoV-2-positive patients) had significantly higher sCr values at discharge than the historical cohort (Table 2).

### 3.3. Clinical Outcomes

**Figure 2:**
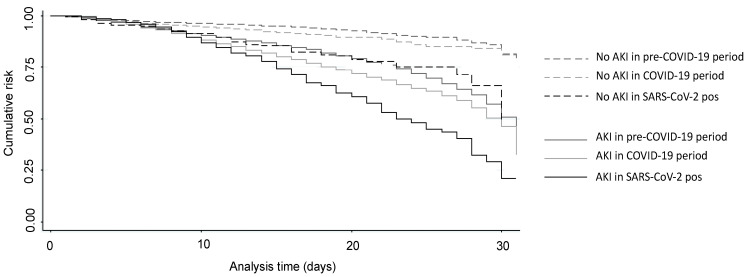
Kaplan–Meier curves of overall survival without death for hospitalized patients (2016–2020) based on the presence of AKI, admission period and/or SARS-CoV-2 infection. Solid lines represent patients who develop AKI, while dashed lines represent patients who did not develop AKI. The grayscale reveals the different admission period and the SARS-CoV-2 infection status (dark gray: pre-COVID-19 period; light gray: SARS-CoV-2-negatives admitted in COVID-19 period; black: SARS-CoV-2 positive admitted in COVID-19 period). Log-rank test *p* < 0.001. Abbreviations: AKI—acute kidney injury; COVID-19—Coronavirus Disease-19; SARS-CoV-2—severe acute respiratory syndrome coronavirus 2.

Regarding hospitalization outcomes, we found that in the COVID-19 period, there was an increased number of patients admitted to ICU, accompanied by a significant increase in the length of hospital stay and intrahospital mortality (Table 4). These data were also confirmed when we separately compared pre-COVID-19 patients with patients admitted in the COVID-19 period according to the positivity to SARS-CoV-2 infection. However, SARS-CoV-2-positive patients presented a further increase in the length of hospital stay and intrahospital mortality, even compared with SARS-CoV-2-negative patients admitted in the same period. Mortality risk factors were investigated in the entire study population by both univariate and multivariate Cox analyses. Univariate analysis showed that age, male sex, ICU admission, septic shock, advanced neoplasia, cardiovascular disease, basal sCr, AKI occurrence, admission period, and SARS-CoV-2 infection were all significantly associated with the mortality rate (Table 5). In the multivariate analysis, after adjustment for clinical and demographic factors, development of AKI, admission in the COVID-19 period, and active SARS-CoV-2 infection remained significantly and independently associated with mortality, both when assessed separately (Model 1) and analyzed in combination (Model 2). We observed that, while in the pre-COVID-19 period AKI development increased the mortality risk (HR 1.34; IC 1.25–1.44), in the COVID-19 period, this risk raised (HR 2.27; IC 2.05–2.50) and further increased when AKI developed in SARS-CoV-2-positive patients (HR 3.8; IC 3.24–4.45) (Figure 2).

## 4. Discussion

We investigated the impact of the COVID-19 pandemic on the epidemiology and outcome of intrahospital AKI in the whole population of patients hospitalized during the COVID-19 pandemic, regardless of SARS-CoV-2 positivity. While previous studies found that patients hospitalized with COVID-19 had a significantly higher incidence of severe AKI compared with controls [12,13], it is not clear whether the COVID-19 pandemic influenced the rate of in-hospital AKI, irrespective of infection status. Overall, we found that AKI was more common and severe in the COVID-19 period than in patients admitted to the same hospital during the four years before the pandemic. As expected, SARS-CoV-2-positive patients presented the highest incidence of AKI, possibly as an expression of the multiple pathological factors that may impair kidney function in this population [14]. However, the increased incidence and severity of AKI resulted significantly also for SARS-CoV-2-negative patients and, indeed, the admission period was independently associated with the occurrence of AKI. Among patients with available previous sCr values, individuals with eGFR < 60 mL/min/1.73 m^2^ were significantly more frequent among SARS-CoV-2-positive patients (41%). Moreover, we found that in SARS-CoV-2-positive patients, there was a significant increase in both AKI occurring in patients with pre-admission eGFR < 60 mL/min/1.73 m^2^ and de novo AKI, while in SARS-CoV-2-negative patients only the incidence of de novo AKI increased. This finding may suggest that intrahospital conditions were probably the prevalent causes of AKI for this subgroup of patients. Regarding AKI-related outcomes, while in agreement with previous studies [15,16], we observed increased AKI-related mortality in SARS-CoV-2-positive patients, we found that this was also true in SARS-CoV-2-negative patients admitted during the COVID-19 period. The differences in AKI development and related outcomes between the patients hospitalized in pre-COVID-19 and COVID-19 periods may recognize many explanations. First of all, patients admitted during the COVID-19 period were older, and more frequently males. Then, they showed fewer chronic comorbidities and were more frequently admitted to ICU areas regardless of SARS-CoV-2 positivity. These observations may indicate that during the pandemic patients resorted to hospitalization mainly for acute needs, as a possible consequence of the fact that many patients with chronic diseases avoided going to the hospital for fear of infection [17]. Interestingly, an exception to this finding was constituted by patients affected by malignancies who, probably due to the severity of the underlying disease, continued to access the hospital also during the pandemic, as proved by the increased prevalence of these patients among SARS-CoV-2-negative patients admitted in the COVID-19 period. Finally, it is not possible to rule out that the different outcomes during the pandemic were also correlated with peculiar pathogenic mechanisms of AKI developing in SARS-CoV-2-infected patients [18]. Moreover, looking again at the kidney function, we found that sCr levels at discharge were significantly higher in patients hospitalized in the COVID-19 period compared to the pre-COVID-19 period. This observation deserves specific consideration since it could potentially impact long-term kidney function, suggesting that patients discharged in the COVID-19 period may need more intensive kidney follow-up to reduce the risk of future CKD development. Apart from considerations on AKI epidemiology, the differences in patients hospitalized in pre-COVID-19 and COVID-19 periods were also evident when we analyzed the general clinical outcomes. Therefore, we found that in the COVID-19 period, the patients presented a longer hospital stay and a significantly higher mortality rate. Remarkably, while we expect these data in SARS-CoV-2-positive patients [19], they were also confirmed in SARS-CoV-2-negative patients. This finding was corroborated by the evidence that the admission period and SARS-CoV-2 positivity independently increased the AKI-related mortality. Our results warrant some consideration. First, differently from most of the other studies that evaluated only the first waves of the pandemic (from March to April 2020), we considered the entire year of 2020 (from January to December) as the COVID-19 period. We made this choice to uniform the data collection, allowing the comparison with the previous years, but also because it is conceivable that changes occurring during the pandemic have had repercussions on the health system and hospital organization throughout 2020. Moreover, there are still doubts about the actual beginning of the COVID-19 pandemic; in Italy, the first cases could be dated back to the beginning of 2020 [20]. Therefore, the inclusion of all of the year 2020 could provide a complete picture of the effects of a pandemic on the health system, even if it does not allow for the evaluation of the potential variations among the different waves [21].

## 5. Limitations

The retrospective monocentric design of our study may represent a limit to the interpretation and generalizability of our findings. Indeed, incidence estimates, mortality rates, and procedures vary among hospitals and countries. Moreover, baseline creatinine was calculated from the lower sCr collected during the hospitalization, which may overestimate or underestimate the incidence of AKI [22]. On the other hand, the appropriate method to evaluate kidney function in hospitalized patients is still a matter of debate since the performance of eGFR formulas in this patient population is poor [23]. Finally, comorbidities and discharge diagnoses were identified using the administrative codes entered in the database, which is subject to bias regarding the use of the incorrect codes [24]. However, these limitations could be partially attenuated since we compared data collected in the same hospital according to a uniform methodology for both periods.

## 6. Conclusions

In conclusion, our data show that during the COVID-19 pandemic, there were significant modifications in AKI epidemiology and outcomes, not only in SARS-CoV-2-positive patients but in the whole hospitalized population. These changes presumably also caused increased costs and workload that, as already observed, could result in health-related, social, and economic problems [25,26]. In this regard, as also suggested by other authors, the COVID-19 pandemic acted as a mass casualty incident, i.e., an event that “overwhelms the local healthcare system, where the number of casualties vastly exceeds the local resources and capabilities in a short period” [27]. The changes in the epidemiology of AKI detected during the COVID-19 pandemic recall the need to adapt the resources dedicated to the prevention and management of the intrahospital AKI in response to health emergencies [28].

## Figures and Tables

**Table 1:** Main clinical characteristics of patients hospitalized in pre-COVID-19 (January 2016–December 2019) and COVID-19 period (January–December 2020).

	Pre-COVID-19 Period	COVID-19 Period			*p*-Value		
		All	SARS-CoV-2 Neg	SARS-CoV-2 Pos	COVID-19 vs. Pre-COVID-19	SARS-CoV-2 Pos vs. SARS-CoV-2 Neg	SARS-CoV-2 Neg vs. Pre-COVID-19
***n***	51,681	10,062	9026	1036			
**Age** (**years**)	69.6 ± 19	71.8 ± 16.8	71.4 ± 17.1	75.1 ± 13.1	<0.0001	<0.0001	<0.0001
**Gender. M%**	46.8	49.2	48.6	55.1	<0.0001	<0.0001	0.002
**Comorbidities, %**							
**Hypertension**	13.9	6	5.7	8.6	<0.0001	<0.0001	<0.0001
**Diabetes**	8.2	3.7	3.6	4.5	<0.0001	0.130	<0.0001
**Heart failure**	8.6	3.4	3.3	4.6	<0.0001	0.023	<0.0001
**Atrial fibrillation**	11.0	5.0	5.0	5.1	<0.0001	0.892	<0.0001
**Malignancies**	7.1	7.6	8.0	3.5	<0.0001	0.113	<0.0001

Differences between the groups were analyzed by independent *t*-tests. Abbreviations: COVID-19—Coronavirus Disease-19; SARS-CoV-2—severe acute respiratory syndrome coronavirus 2.

**Table 2:** Kidney function and AKI epidemiology of patients hospitalized in pre-COVID-19 period (January 2016–December 2019) and COVID-19 period (January–December 2020).

	Pre-COVID-19 Period	COVID-19 Period			*p*-Values		
		All	SARS-CoV-2 Neg	SARS-CoV-2 Pos	COVID-19 vs. Pre-COVID-19	SARS-CoV-2 Pos vs. SARS-CoV-2 Neg	SARS-CoV-2 Neg vs. Pre-COVID-19
***n***	51,681	10,062	9026	1036			
**sCr at admission (µmol/L)**	94.2 ± 60.7	96.8 ± 61.6	95.9 ± 62.5	100.3 ± 61.6	<0.0001	0.0211	0.005
**AKI incidence. *n* (%)**	13,377 (25.9)	3184 (31.7)	2750 (30.5)	436 (42.2)	<0.0001	<0.0001	<0.0001
**AKI staging, (%)**					<0.0001	<0.0001	<0.0001
**- stage 1**	19.8	22.9	22.1	30.3			
**- stage 2**	4.3	5.8	5.6	7.2			
**- stage 3**	1.8	3.0	2.8	4.6			
**Patients with previous** **sCr available, *n* (%)**	18,785 (36)	3257 (32)	2754 (27)	503 (48)			
**eGFR < 60 mL/min/1.73 m^2^,** ***n* (%)**	6136 (32.7)	1095 (33.6)	885 (32.1)	210 (41.7)	0.284	<0.0001	0.580
**AKI/** **eGFR < 60 mL/min/1.73 m^2^** **, *n* (%)**	2924 (15.6)	566 (17.4)	448 (16.3)	118 (23.5)	0.009	<0.0001	0.344
**“De novo” AKI, *n* (%)**	2369 (12.6)	537 (16.5)	439 (15.9)	98 (19.5)	<0.0001	0.049	<0.0001
**sCr at discharge (µmol/L)**	89.8 ± 52.8	93.3 ± 59.8	92.4 ± 59	102.1 ± 68.6	<0.0001	<0.0001	<0.0001

“De novo” AKI was defined as AKI occurring in patients with pre-admission eGFR ≥ 60 mL/min/1.73 m^2^. Differences between the groups were analyzed by independent *t*-tests. Abbreviations: eGFR—estimated glomerular filtration rate; sCr—serum creatinine; AKI—acute kidney injury; COVID-19—Coronavirus Disease-19; SARS-CoV-2—severe acute respiratory syndrome coronavirus 2.

**Table 3:** Logistic models for the development of acute kidney injury in hospitalized patients.

	Univariate			Multivariate Model		
Risk Factors	OR	95% CI	*p*	OR	95% CI	*p*
**Gender** (**male**)	1.08	1.05–1.07	<0.0001	1.06	1.01–1.10	0.006
**Age**	1.04	1.04–1.12	<0.0001	1.03	1.03–1.03	<0.0001
**Comorbidities**						
**CVD**	3.06	2.88–3.25	<0.0001	1.85	1.73–1.98	<0.0001
**Diabetes**	1.28	1.20–1.37	<0.0001	0.99	0.92–1.06	0.852
**Basal sCr**	2.57	2.45–2.68	<0.0001	2.69	2.56–2.83	<0.0001
**Medical ward**	ref			ref		
**ICU stay**	1.74	1.62–1.86	<0.0001	2.4	2.21–2.6	<0.0001
**Length of stay** (**day**)	1.13	1.13–1.13	<0.0001	1.13	1.13–1.14	<0.0001
**SARS-CoV-2 infection**	2.09	1.84–2.37	<0.0001	1.30	1.12–1.5	<0.0001
**Admission in pre-COVID-19**	ref			ref		
**Admission in COVID-19**	1.25	1.19–1.32	<0.0001	1.18	1.12–1.25	<0.0001

Abbreviations: CVD—cardiovascular disease; sCr—serum creatinine; ICU—intensive care unit; COVID-19—Coronavirus Disease-19; SARS-CoV-2—severe acute respiratory syndrome coronavirus 2.

**Table 4:** Clinical outcomes of patients hospitalized in pre-COVID-19 period (January 2016–December 2019) and COVID-19 period (January–December 2020).

	Pre-COVID-19 Period	COVID-19 Period			*p*-Values		
		All	SARS-CoV-2 Neg	SARS-CoV-2 Pos	COVID-19 vs. Pre-COVID-19	SARS-CoV-2 Pos vs. SARS-CoV-2 Neg	SARS-CoV-2 Neg vs. Pre-COVID-19
***n***	51,681	10,062	9026	1036			
**Mortality rate, %**	7.2	12.2	10.7	24.9	<0.0001	<0.0001	<0.0001
**ICU admission, %**	5.3	8.8	9.0	7.3	<0.0001	0.100	<0.0001
**Length of stay (days)**	9.5 ± 6.7	10.7 ± 7.1	10.4 ± 6.9	13.6 ± 7.4	<0.0001	<0.0001	<0.0001
**Length of stay > 15 days, %**	21.7	26.0	24.1	42.2	<0.0001	<0.0001	<0.0001

Abbreviations: ICU—intensive care unit; COVID-19—Coronavirus Disease-19; SARS-CoV-2—severe acute respiratory syndrome coronavirus 2.

**Table 5:** Univariate and multivariate Cox regression analyses for intrahospital mortality in hospitalized patients between 2016 and 2020.

	Univariate			Multivariate Model 1			Multivariate Model 2		
Risk Factors	HR	95% CI	*p*	HR	95% CI	*p*	HR	95% CI	*p*
**Gender (male)**	1.05	0.999–1.11	0.103	1.14	1.08–1.21	<0.0001	1.14	1.08–1.21	<0.0001
**Age**	1.04	1.04–1.04	<0.0001	1.03	1.03–1.04	<0.0001	1.03	1.03–1.04	<0.0001
**Comorbidities**									
**CVD**	3.1	2.90–3.31	<0.0001	2.41	2.25–2.58	<0.0001	2.41	2.25–2.6	<0.0001
**Sepsis**	9.9	9.21–10.6	<0.0001	3.2	2.99–3.49	<0.0001	3.24	3.0–3.5	<0.0001
**Advanced Neoplasia**	2.08	1.78–2.43	<0.0001	3.05	2.61–3.57	<0.0001	3.05	2.61–3.58	<0.0001
**Basal sCr**	1.24	1.22–1.25	<0.0001	1.19	1.17–1.21	<0.0001	1.19	1.17–1.21	<0.0001
**Medical ward**	Ref								
**ICU stay**	1.51	1.37–1.66	<0.0001	1.94	1.76–2.15	<0.0001	1.94	1.76–2.15	<0.0001
**Admission in COVID-19 period**	1.49	1.39–1.59	<0.0001	1.60	1.49–1.73	<0.0001	-		
**SARS-CoV-2 infection**	2.17	1.91–2.46	<0.0001	1.68	1.46–1.93	<0.0001	-		
**Overall AKI**	2.55	2.40–2.71	<0.0001	1.39	1.3–1.48	<0.0001	-		
**No AKI in pre-COVID-19**	Ref.						Ref.		
**AKI in pre-COVID-19**	2.56	2.39–2.74	<0.0001				1.34	1.25–1.44	<0.0001
**No AKI in COVID-19 period**	1.35	1.19–1.52	<0.0001				1.47	1.30–1.67	<0.0001
**AKI in COVID-19 period**	3.33	3.03–3.67	<0.0001				2.27	2.05–2.50	<0.0001
**No AKI in SARS-CoV-2 pos**	2.49	1.99–3.12	<0.0001				2.51	2.0–3.15	<0.0001
**AKI in SARS-CoV-2 pos**	5.12	4.37–5.99	<0.0001				3.80	3.24–4.45	<0.0001

Multivariate Model 1 includes gender, age, comorbidities, ICU stay, admission in COVID-19 period, SARS-CoV-2 infection and overall AKI prevalence. Multivariate Model 2 includes gender, age, comorbidities, ICU stay, and the presence or the absence of AKI in the different periods (pre-COVID-19 or COVID-19). Abbreviations: CVD—cardiovascular disease; sCr—serum creatinine; ICU—intensive care unit; AKI—acute kidney injury; COVID-19—Coronavirus Disease-19; SARS-CoV-2—severe acute respiratory syndrome coronavirus 2.

## Data Availability

The datasets used and/or analyzed during the study are available from the corresponding author upon reasonable request.

## Supplementary Materials

The following supporting information can be downloaded at: https://www.mdpi.com/article/10.3390/jcm11123349/s1, Table S1: Main discharge diagnosis of patients hospitalized in pre-COVID-19 (January 2016–December 2019) and COVID-19 period (January–December 2020); Table S2: AKI incidence and staging during all the observational period.
