# Supplementary material for: Changes of Acute Kidney Injury Epidemiology during the COVID-19 Pandemic: A Retrospective Cohort Study
Source: J Clin Med. 2022 Jun 10;11(12):3349. doi: 10.3390/jcm11123349 (PMC9225342; doi:10.3390/jcm11123349)
Supplement: Supplementary file 1 [file jcm-11-03349-s001.zip › jcm-1728157-supplementary.pdf]

CHANGES OF IN-HOSPITAL ACUTE KIDNEY INJURY EPIDEMIOLOGY AND OUTCOMES DURING COVID-19 PANDEMIC: A RETROSPECTIVE COHORT STUDY

SUPPLEMENTAL MATERIALS

Supplementary Table S1. Main discharge diagnosis of patients hospitalized in pre-COVID-19 (Jan 2016-Dec 2019) and COVID-19 period (Jan-Dec 2020)

	Pre-COVID-19 period	COVID-19 period			p-values		
		All	SARS-CoV-2 neg	SARS-CoV-2 pos	COVID19 vs PreCOVID19	SARS-CoV-2 pos vs SARS-Cov-2 neg	SARS-CoV-2 neg vs Pre-COVID-19
N.	51681	10062	9026	1036			
Discharge diagnosis, %							
Respiratory diseases [a]	9.93	20.22	15.91	55.02	<0.0001	<0.0001	<0.0001
Infectious diseases [a]	1.17	1.11	0.8	0.39	<0.0001	0.147	0.002
Cardiovascular diseases	22.68	21.35	22.04	12.55	<0.0001	<0.0001	0.176
Gastrointestinal disease	11.31	10.38	10.75	5.79	0.002	<0.0001	0.119
Urogenital diseases	5.18	4.57	4.69	2.90	0.004	0.008	0.048
CNS Diseases	6.81	6.15	6.47	2.51	0.006	<0.0001	0.239
Mental disease	7.29	5.19	5.44	2.32	<0.0001	<0.0001	<0.0001
Endocrine disorders	2.14	1.43	1.48	0.77	<0.0001	0.066	<0.0001
Hematological disorders	1.52	1.24	1.21	1.35	0.024	0.690	0.023
Pregnancy complications	1.56	1.47	1.07	0.19	<0.0001	0.006	<0.0001
Bone disease	1.14	1.09	0.91	0.1	0.006	0.006	0.054
Poisoning	14.60	14.48	14.38	5.21	0.002	<0.0001	0.590

[a] In Italy, SARS-CoV-2 infection was codified as a respiratory disease instead of an infectious disease.

Abbreviations: CNS, central nervous system; COVID-19, Coronavirus Disease-19 SARS-CoV-2, severe acute respiratory syndrome coronavirus 2

Supplementary Table S2. AKI incidence and staging during all the observational period

	Year (Jan-Dec)	no AKI n, (%)	AKI stage 1 n, (%)	AKI stage 2 n, (%)	AKI stage 3 n, (%)	Total
Pre-COVID-19 period	**2016**	11,270	2,761	625	282	14,938
		(75.45)	(18.48)	(4.18)	(1.89)	100.00
	**2017**	10,349	2,670	596	260	13,875
		(74.59)	(19.24)	(4.30)	(1.87)	100.00
	**2018**	7,700	1,948	402	145	10,195
		(75.53)	(19.11)	(3.94)	(1.42)	100.00
	**2019**	8,971	2,824	612	266	12,673
		(70.79)	(22.28)	(4.83)	(2.10)	100.00
COVID-19 period	**2020**	6,871	2,308	581	302	10,062
		(68.29)	(22.94)	(5.77)	(3.00)	100.00
	**Total**	45,161	12,511	2,816	1,255	61,743
		(73.14)	(20.26)	(4.56)	(2.03)	100.00

Abbreviations: AKI, acute kidney injury; COVID-19, Coronavirus Disease-19
